# Endoscopic ultrasound combined with cholangioscopy in the diagnosis and treatment of pancreatic arteriovenous malformation-related repeated duodenal bleeding

**DOI:** 10.1055/a-2665-8010

**Published:** 2025-08-21

**Authors:** Shan-Shan Hu, Shan Lei, Jie Hou, Wei-Hui Liu

**Affiliations:** 1 89669 Department of Gastroenterology and Hepatology, Sichuan Provincial Peopleʼs Hospital, School of Medicine, University of Electronic Science and Technology of China, Chengdu, China

Pancreatic arteriovenous malformation (P-AVM) is a rare vascular anomaly, typically presenting with gastrointestinal bleeding, abdominal pain, jaundice, or portal hypertension. Digital subtraction angiography remains the conventional diagnostic modality for this condition 1 2 3 4 . Recently, our team successfully diagnosed a case of P-AVM utilizing a novel diagnostic approach – endoscopic ultrasound (EUS) combined with cholangioscopy – and performed localized therapy, resulting in favorable clinical outcomes.

A 52-year-old male patient was admitted for the management of acute cholangitis. During EUS, thickening of the bile duct wall and gas accumulation within the lumen were observed. In the region of the pancreatic head, a honeycomb-like hypoechoic lesion encasing the pancreatic segment of the common bile duct was identified ( Fig. 1 ). Color Doppler imaging revealed the lesion to comprise varicose veins, with partial venous drainage into the portal vein. Spectral Doppler analysis further demonstrated pulsatile arterial waveforms, supporting the diagnosis of P-AVM ( Fig. 2 ). Subsequent cholangioscopic examination identified a deep, excavated ulcer adjacent to the duodenal papilla, which had resulted in a choledochoduodenal fistula with continuous leakage of bile and pancreatic juice into the ulcer bed. Exposed vessels were visible at the ulcer base, and active hemorrhage from vascular stumps was successfully managed with topical hemostatic agents ( Fig. 3 ). The cholangioscope was advanced through the fistula into the bile duct, enabling direct visualization of the roughened and hyperemic mucosa. It was concluded that P-AVM had led to ischemia and necrosis of the intestinal wall, thereby inducing duodenal ulceration and fistula formation ( Fig. 4 ). The corrosive effects of bile and pancreatic juice further exacerbated this pathological cycle. To mitigate further damage, separate biliary and pancreatic duct drainage tubes were placed, effectively isolating the ulcer base from digestive secretions ( Fig. 5 , Video 1 ).

**Fig. 1:**
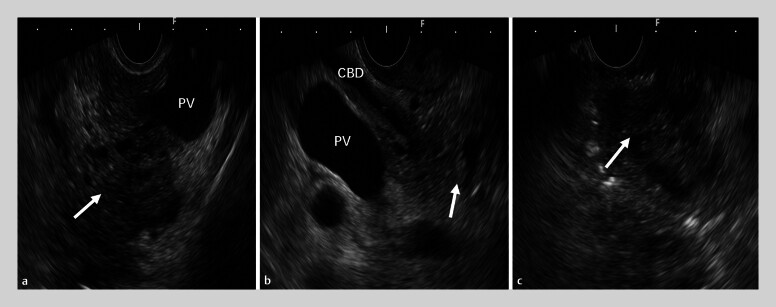
EUS revealed a cystic lesion in the pancreatic head. **a** During the first station examination, a cystic lesion was visualized in the pancreatic head region (indicated by the arrow). **b** During the second station examination, bile duct wall thickening and intraluminal gas accumulation were observed, along with cystic lesions encasing the pancreatic segment of the common bile duct (indicated by the arrow). **c** During the third station examination, cystic components were noted converging into the portal vein (indicated by the arrow).

**Fig. 2:**
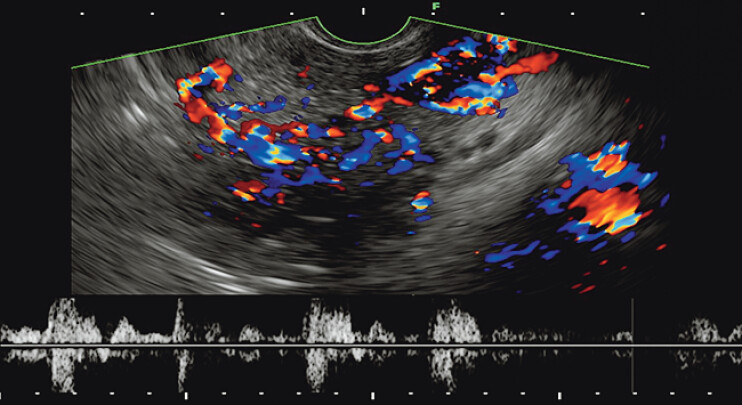
The application of color Doppler imaging contributed to the identification of P-AVM. Color Doppler imaging revealed tortuous and dilated blood vessels. Spectral Doppler analysis demonstrated arterial pulsatile flow characteristics.

**Fig. 3:**
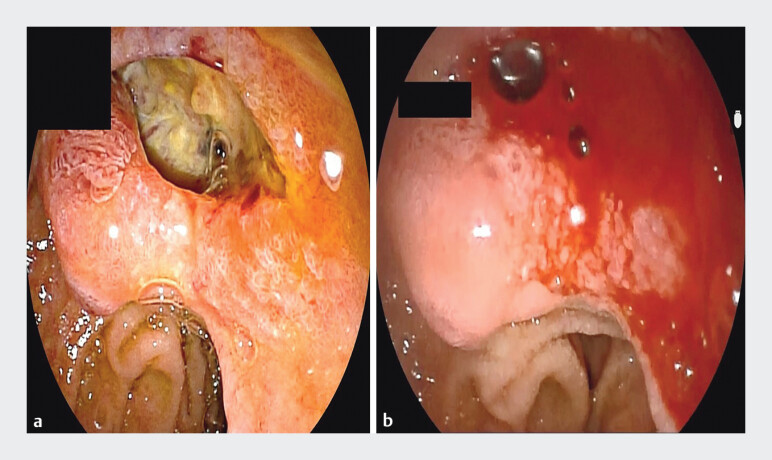
Ulcer adjacent to the duodenal papilla. **a** Exposed blood vessels were visible at the ulcer center. **b** Sudden hemorrhage from exposed vasculature.

**Fig. 4:**
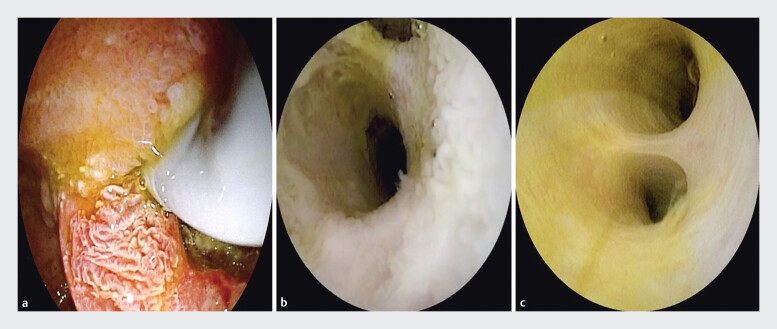
Cholangioscopic visualization of a choledochoduodenal fistula. **a** The cholangioscope was advanced into the common bile duct through the fistulous opening. **b** The orifices of both the bile and pancreatic ducts were identified. **c** The bile duct mucosa appeared rough and congested.

**Fig. 5:**
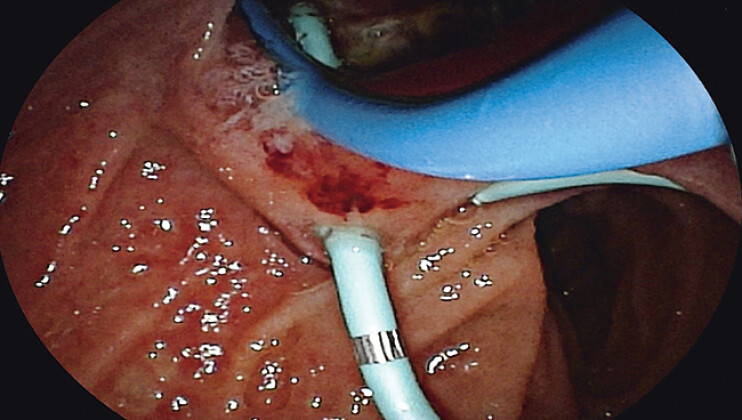
Placement of biliary and pancreatic duct drainage tubes.

Although P-AVM is diagnosed through angiographic imaging, this case highlights an innovative diagnostic and therapeutic strategy by integrating EUS with cholangioscopy.Video 1

Although P-AVM is most commonly diagnosed through angiographic imaging, this case highlights an innovative diagnostic and therapeutic strategy by integrating EUS with cholangioscopy. This approach enabled early identification and intervention, demonstrating considerable clinical value and potential for broader clinical application.

Endoscopy_UCTN_Code_CCL_1AB_2AZ_3AD

## Notes

Endoscopy E-Videos https://eref.thieme.de/e-videos
*E-Videos* is an open access online section of the journal *Endoscopy* , reporting on interesting cases and new techniques in gastroenterological endoscopy. All papers include a high-quality video and are published with a Creative Commons CC-BY license. Endoscopy E-Videos qualify for HINARI discounts and waivers and eligibility is automatically checked during the submission process. We grant 100% waivers to articles whose corresponding authors are based in Group A countries and 50% waivers to those who are based in Group B countries as classified by Research4Life (see: https://www.research4life.org/access/eligibility/ ). This section has its own submission website at https://mc.manuscriptcentral.com/e-videos .
